# Children with Autism Spectrum Disorder of All Ages, Levels of Symptom Severity and General Cognitive Ability Display Low Processing Speed Index Scores Warranting Special Educational Assistance

**DOI:** 10.1007/s10803-021-05249-5

**Published:** 2021-08-27

**Authors:** M. Linnenbank, R. Feldmann, G. Schulte-Körne, S. Beimdiek, E. Strittmatter

**Affiliations:** 1 Department of Pediatrics, University of Münster, Munster, Germany; 2 Department of Child and Youth Psychiatry, Ludwig-Maximilians University of Munich, Munich, Germany; 3 Children’s Healthcare Center “Haus Walstedde”, Drensteinfurt, Germany; 4 University Hospital Münster, Albert-Schweitzer-Campus 1, 48149 Munster, Germany

**Keywords:** Autism spectrum disorder, Children, Processing speed index, WISC-IV, Special educational assistance, Academic achievement

## Abstract

The processing speed index (PSI) of the Wechsler intelligence scale for children (WISC-IV) has been found to predict a child's level of academic functioning. The consistently reported PSI weakness in children with autism spectrum disorder (ASD) therefore warrants special assistance and attempts at compensation for the disadvantages associated with these children's low PSI. We investigated the association of PSI scores with age, general cognitive ability [as measured by full-scale IQ (FSIQ)], symptom severity and discrepancy between the WISC-IV indices verbal comprehension (VCI) and perceptual reasoning (PRI) in 101 school children with ASD. The PSI weakness in children with ASD was not related to age, FSIQ, VCI-PRI discrepancy or any of the symptom measures. These findings suggest that school children with ASD independent of their age, level of cognitive ability, VCI-PRI profile and most notably independent of their symptom severity should be entitled to special assistance and compensation in educational settings.

## Introduction

Intellectual functioning is one of the most extensively studied aspects in children with autism spectrum disorder (ASD; Oliveras-Rentas et al., 2012). In the diagnostic process of ASD, it is usually assessed by means of the Wechsler intelligence scale for children (WISC; Wechsler, 2003). In previous research, WISC profiles in children with ASD have been found to be marked by a higher degree of discrepancy between indices than is the case in typically developing children (Joseph et al., 2002). The verbal comprehension (VCI) and perceptual reasoning (PRI) indices yield the highest mean scores, while the processing speed index (PSI) is typically the lowest score in samples of children with ASD (Foley-Nicpon et al., 2012; Mayes & Calhoun, 2008; Oliveras-Rentas et al., 2012; Styck et al., 2019; Zander & Dahlgren, 2010). The working memory index (WMI) has been found to take intermediate levels in some studies (e.g. Styck et al., 2019) and low levels similar to the PSI in other studies (e.g. Rabiee et al., 2019). The PSI has been consistently found to constitute not only a relative weakness (compared to other WISC indices) but also an absolute weakness (compared to the norm) in children with ASD (Oliveras-Rentas et al., 2012). Assessment of intellectual functioning in children with ASD by means of WISC is commonly made in order to inform schooling matters or to predict academic achievement. To this end, the PSI appears to be of particular interest as it has been shown to confer considerable predictive value with regard to different aspects of a child’s functioning in educational settings: children with low PSI scores display greater difficulties in “soft skills” required in school settings, like “getting started on a task/activity” according to teacher reports (Lundervold et al., 2011). Mayes and Calhoun (2007) found that processing speed weaknesses tend to occur together with difficulties in graphomotor ability and attention. Furthermore, children with lower processing speed attained significantly lower achievement scores in math, reading and written expression than it would have been expected based on their FSIQ. The influence of processing speed on academic achievement thus extends beyond general cognitive ability (Mayes & Calhoun, 2007; Rohde & Thompson, 2007). Moreover, PSI (and VCI) scores were found to specifically predict the extend of educational assistance needed by a child. The lower a child’s PSI score, the more hours the child spent with the special education assistant (Grimm et al., 2015). Similarly, there is evidence that processing speed plays an important role in the development of executive functioning (Fry & Hale, 2000; Gordon et al., 2018; McAuley & White, 2011). Together, these findings illustrate the impact of processing speed capacities on a child’s functioning in educational settings. They also suggest that children with low PSI scores—among them children with ASD—are in need of special support in these settings and should be entitled to assistance and measures that compensate for the disadvantages their processing speed weakness confers.

In light of these striking results indicating that such special support for children with ASD is highly warranted, the following central question arises: Is the PSI weakness (and therefore the required and justified degree of special assistance and compensation for disadvantage) in children with ASD associated with factors like age, general cognitive ability and degree of symptom manifestation? In previous research, different studies have examined how processing speed is related to autism symptomatology and associated difficulties—yielding inconsistent results: while some found processing speed to be correlated with communication symptoms, motor skills, and daily living skills (Hedvall et al., 2013; Oliveras-Rentas et al., 2012), others found no relationship between the PSI and autistic symptoms (Mouga et al., 2016; Rabiee et al., 2019). One study that may provide some hints concerning the influence of general cognitive ability (as measured by WISC FSIQ) on the PSI compared ASD children with (FSIQ < 70) and without intellectual disability (Mouga et al., 2016). In this study, the PSI weakness was found to be more pronounced in the group with intellectual disability. However, more research is needed to elucidate the association between PSI and general cognitive ability in children *without* intellectual disability. The only study to our knowledge that has examined the relationship between PSI and age in ASD assessed ASD individuals between six and 39 years of age and found no age-related changes in PSI scores over time (Travers et al., 2014).

The aim of the present study is to investigate whether age, general cognitive ability (as measured by WISC-IV FSIQ), and symptom severity [as measured by the autism diagnostic observation schedule (ADOS; Lord et al., 1989) and the autism diagnostic interview—revised (ADI-R; Lord et al., 1989, 1994)] are related to PSI scores in our sample of 101 children with thoroughly diagnosed ASD. In addition, the role of IQ split, that is, an uneven ability profile of discrepantly higher VCI than PRI or vice-versa, in relation to PSI scores will be examined. A significant IQ split between verbal and non-verbal ability has been found to be associated with ASD symptomatology (Ankenman et al., 2014; Black et al., 2009; Joseph et al., 2002), fine motor skills (Yu et al., 2018), working memory, planning, organization and monitoring abilities (Kalbfleisch & Loughan, 2012) in children with ASD. On the basis of these findings, a verbal-nonverbal discrepancy has been proposed to constitute a phenotypic marker pointing to etiologically different subtypes of autism (Joseph et al., 2002). Consequently, our study will investigate whether PSI scores in ASD children are related to presence and/or direction of IQ discrepancy. The results will help elucidate whether the degree of special assistance and compensation for disadvantages conveyed by processing speed weaknesses is warranted for all school children with ASD or whether it should vary with age, symptom severity, general cognitive ability or IQ split. The results will also valuably contribute to raising awareness for the needs of children with ASD in teachers, parents and clinicians. Finally, in the development and application of treatment options for children with ASD, it is important to know whether low processing speed needs to be taken into account regardless of the degree of symptom manifestation.

## Methods

### Patients

Data were obtained between May 2015 and April 2017 from a sample of German children who were out-patients of a specialized day-unit center for ASD. The initial sample (*N* = 184) included all patients between six and 16 years of age with a diagnosis of Autism Spectrum Disorder according to the Diagnostic and Statistical Manual of Mental Disorders, fifth version (DSM-5; American Psychiatric Association, 2013), for whom complete data from the ADOS (first version) and the ADI-R and the German version of the WISC-IV (Petermann & Petermann, 2010) were available. Each child was thoroughly diagnosed by a skilled professional inter-disciplinary team of psychiatrists and psychologists specialized in ASD for many years in a series of seven diagnostic sessions (including ‘diagnostic play’ sessions) and in close exchange with the child’s schooling institution. From this sample, patients with FSIQ < 70 (*n* = 51) were excluded in order to avoid confounding influence of intellectual disability in the measurement of cognitive functioning. Apart from that, this study aimed to specifically investigate the necessity of special support for children with ASD without intellectual disability who attend regular or special-needs schools. The needs of children with accompanying intellectual disability are usually more obvious and there tends to be more awareness. In line with these exclusion criteria, all participants had a FSIQ of 70 or higher and displayed fluent language. Therefore, Modules 1 and 2 of the ADOS, which are designed for children with impaired language were not applied. Only very few children (*n* = 6) received Module 4. In order to keep the data set as homogenous as possible, these children were excluded, so that all children who remained in the sample had received Module 3. Furthermore, all cases of co-morbid psychiatric diseases (*n* = 26), comprising psychotic disorders, depression, oppositional defiant disorders, anxiety and tic disorders, were excluded from the study. Children with co-morbid Attention-Deficit/Hyperactivity Disorder (ADHD; *n* = 30) were not excluded. Because the proportion of ASD patients with co-morbid ADHD is very high, and because of the high phenotypical overlap between ASD and ADHD children (Sinzig et al., 2009), it was decided to proceed as follows with this group of patients: independent samples t-tests were conducted in order to compare individuals with and without accompanying ADHD. While children with ADHD without ASD have been found to be less likely to display a PSI weakness than children with ASD (Mayes & Calhoun, 2007), the two groups with ASD with and without ADHD did not differ on any of the investigated variables (age, IQ split, ADOS, ADI-R, and WISC-IV scores) at a significance level of *p* = 0.05. Therefore, patients with a co-diagnosis of ADHD were included in the study. After the application of exclusion criteria, a sample of *N* = 101 patients, 91 boys and 10 girls, remained. Because of the small number of female participants in our sample (*n* = 10), an analysis for equality of means between the two groups (males and females) was conducted using a Welch’s t-test. As no differences were found between boys and girls on any of the investigated variables at a significance level of *p* = 0.05, female participants were included in the study.

### Measures

#### The Autism Diagnostic Observation Schedule (1st ed.; ADOS)

The ADOS (Lord et al., 1989) is a semi-structured interview that evokes different naturalistic situations that demand social and communicative reactions from the child that are subsequently coded by the clinician. The ADOS yields separate scores for ‘social interaction’ (0–14 points), ‘communication’ (0–8 points), and ‘restricted and/or repetitive behaviors’ (0–8 points). The higher the score, the more severely impaired a child’s functioning is on that domain.

#### The Autism Diagnostic Interview—Revised (ADI-R)

The ADI-R (Lord et al., 1994) is a structured diagnostic interview with the child’s caregiver, that explores the areas of ‘reciprocal social interaction’ (with scores ranging from 0 to 30), ‘communication and language’ (scores ranging from 0 to 26), and ‘restricted and repetitive behaviors’ (scores ranging from 0 to 16) and provides a separate score for each of these domains. Higher domain scores indicate more severe symptoms.

#### The Wechsler Intelligence Scale for Children (4th ed.; WISC-IV)

The WISC (Wechsler, 2003) is the most widely used instrument to measure intellectual functioning in children. The fourth version of the WISC comprises ten mandatory sub-tests which can be clustered into four composite index scores: The verbal comprehension index (VCI), the perceptual reasoning index (PRI), the working memory index (WMI) and the processing speed index (PSI). There are also five optional subtests that can be used to supplement for one of the others if it cannot be administered. However, in order to keep the dataset as homogenous as possible, all scores used in this study were obtained using the ten core sub-tests, the results of which combine to yield an index of general cognitive ability, the full-scale intelligence quotient (FSIQ). In this study, the German version of the WISC-IV was used (Petermann & Petermann, 2010).

### Data Interpreted in this Study

ADI-R (Lord et al., 1994) and ADOS (1st ed.; Lord et al., 1989) were conducted for each child in the context of the diagnostic process. For our study, we analyzed the corresponding domain scores of *Communication* (ADOS-A and ADI-B), *Reciprocal social interaction* (ADOS-B and ADI-A), and *Stereotypic/repetitive behavior* (ADOS-D and ADI-C). Many of the previous studies employing ADOS and ADI-R data merged the corresponding domain scores of the two tests into one score per domain. However, our analysis of Pearson’s correlations between ADOS and ADI-R scores showed that the correlations between the two corresponding scores of each domain were either non-significant or very small (see Table 3 for correlation coefficients and *p*-values). Therefore, ADOS and ADI-R scores were considered separately in statistical analysis.

Of the WISC-IV data, FSIQ was used to assess general cognitive ability. PRI and VCI were used in order to cluster the groups according to IQ split, and the PSI was used as outcome variable.

### Ethics

The study was approved by the ethics committee.

### Statistical Analysis

Data were analyzed using the SPSS Statistics program, version 26 (IBM, Amonk, USA). The ASD group’s PSI score was compared to the norm (*M* = 100, *SD* = 15) with a one-sample t-test. A within-subject ANOVA calculation was conducted in order to compare the PSI to the other indices within the ASD sample. Subsequently, this within-subject analysis was repeated for two (split-half) groups, one with higher (≥ 100) and one with lower (≤ 99) FSIQ, separately, in order to investigate whether the profiles differ between children with higher and lower general cognitive ability. The association between age and symptom severity and the PSI was assessed by means of Pearson’s correlations. Of the ADOS and ADI-R, raw scores were used for this calculation as all participants received the same modules and tests. Significance levels were adjusted according to the Bonferroni method in order to correct for multiple comparisons. Finally, the sample was divided into three groups according to IQ split: (1) children with discrepantly higher (≥ 11 points difference) VCI than PRI, (2) children with discrepantly higher (≥ 11 points difference) PRI than VCI, and (3) children with approximately equal VCI and PRI (< 11 points difference). The criterion for discrepancy of 11 points was adapted from a previous study (Black et al., 2009), in which the 11-point difference had been determined based on IQ discrepancies that reach statistical significance at *p* = 0.05 according to three different measures of intelligence (WISC-III, WISC-IV, and the Wechsler Abbreviated Scale of Intelligence (WASI; Wechsler, 1999). These IQ split groups were then compared by means of a univariate ANOVA calculation.

## Results

### Descriptive Statistics

Of our sample of 101 children between six and 16 years of age, 90.1% (*n* = 91) were male. Co-morbid ADHD was diagnosed in 30 children (29.7% of the sample), i.e. in 30% of the boys (*n* = 29) and in 10% of the girls (*n* = 1). All mean scores with corresponding standard deviations and ranges of FSIQ, indices of the WISC-IV and all domains of the ADOS and the ADI-R can be found in Table 1.

**Table 1:** Descriptive characteristics of the autism spectrum disorder sample as a whole

Variable	*M*	*SD*	Minimum	Maximum
Age	10.63	2.72	6	16
WISC-IV PRI	104.87	14.82	71	144
WISC-IV VCI	103.59	14.84	71	142
WISC-IV WMI	96.63	12.51	71	144
WISC-IV PSI	91.68	13.26	56	131
WISC-IV FSIQ	99.84	13.23	70	143
Communication				
ADOS (A)	4.01	1.75	0	7
ADI-R (B)	12.21	3.99	3	22
Reciprocal social interaction				
ADOS (B)	6.71	2.54	1	13
ADI-R (A)	14.65	5.18	1	25
Stereotypic/repetitive behavior				
ADOS (D)	0.78	1.07	0	6
ADI-R (C)	3.73	2.14	0	10

*N* = 101, *M* mean, *SD* standard deviation, *WISC-IV* Wechsler intelligence scale for children, fourth version, *PRI* perceptual reasoning index, *VCI* verbal comprehension index, *WMI* working memory index, *PSI* processing speed index, *FSIQ* full-scale intelligence quotient, *ADOS* autism diagnostic observation scale, *ADI-R* autism diagnostic interview—revised

### The WISC-IV Index Scores

The PSI was significantly lower in the group with ASD than the defined norm, *t*(100) = − 6.30, *p* < 0.001. The repeated measures ANOVA indicated significant differences between the indices within the ASD group, *F*(4,400) = 28.69, *p* < 0.001. Pairwise comparisons confirmed that the PSI was significantly lower (*p* < 0.001) than VCI and PRI. The same was found in the split-half groups with higher (≥ 100) FSIQ, *F*(4,192) = 27.61, *p* < 0.001, and lower (≤ 99) FSIQ, *F*(4,204) = 8.31, *p* < 0.001. The results of this part of the statistical analysis are shown in Table 2.

**Table 2:** WISC-IV indices for split-half groups with higher and lower FSIQ with *p* values of pairwise comparisons with the PSI and effect size for each index

Index score	FSIQ ≥ 100^a^				FSIQ ≤ 99^b^			
	*M*	*SD*	*p*	Effect size	*M*	*SD*	*p*	Effect size
VCI	113.65	11.22	< 0.001***	0.567	94.12	11.14	0.008**	0.331
PRI	114.31	11.20	< 0.001***	0.583	95.98	12.12	0.001*	0.383
WMI	101.82	13.17	0.631	0.161	91.75	9.67	0.081	0.256
PSI	97.71	11.93			86.00	11.96		
FSIQ	110.53	8.63	< 0.001***	0.524	89.77	7.74	0.165	0.161

*M* mean, *SD* standard deviation, *WISC-IV* Wechsler intelligence scale for children, fourth version, *PRI* perceptual reasoning index, *VCI* verbal comprehension index, *WMI* working memory index, *PSI* processing speed index, *FSIQ* full-scale intelligence quotient, *ADOS* autism diagnostic observation scale, *ADI-R* autism diagnostic interview—revised

^a^*n* = 49

^b^*n* = 52

*p < 0.05

**p < 0.01

***p < 0.001

### Correlation Between PSI Scores and Age, ADOS and ADI-R Measures

Mean scores, SDs, *p*-values and Pearson’s correlation coefficients can be found in Table 3. Of all ADOS and ADI-R domains, ADOS communication was the only one that correlated significantly with the PSI, *r*(99) = − 0.241, *p* = 0.015. However, significance of this correlation did not bear the correction for multiple comparisons. After the application of the Bonferroni correction, neither age nor any of the ADOS or ADI-R domain scores showed a significant correlation with the PSI.

**Table 3:** Pearson’s correlation coefficients

Variable	1	2	3	4	5	6	7	8
1. PSI	–							
2. Age	− 0.025	–						
3. Communication—ADOS (A)	− 0.241	0.137	–					
4. Communication—ADI-R (B)	− 0.038	0.083	0.145	–				
5. Reciprocal social interaction—ADOS (B)	− 0.122	0.083	0.550**	0.143	–			
6. Reciprocal social interaction—ADI-R (A)	− 0.028	0.153	0.060	0.509**	0.263	–		
7. Stereotypic/repetitive behavior—ADOS (D)	− 0.057	− 0.079	0.313*	0.098	0.338*	− 0.110	–	
8. Stereotypic/repetitive behavior—ADI-R (C)	− 0.036	− 0.010	0.161	0.346**	− 0.006	0.252	0.283*	–

*PSI* processing speed index, *ADOS* autism diagnostic observation scale, *ADI-R* autism diagnostic interview—revised

*p < 0.007 (α adjusted according to Bonferoni)

**p < 0.001

### Association Between IQ Split and PSI Scores

The univariate ANOVA analysis yielded no significant differences in PSI scores between the three IQ split groups *F*(2,98) = 0.66, *p* = 0.519. Table 4 displays frequency, mean PSI and mean FSIQ for the three IQ split groups.

**Table 4:** Descriptive characteristics of the IQ split groups

IQ split profile	*n*	%	PSI		FSIQ	
			*M*	*SD*	*M*	*SD*
VCI = PRI^a^	45	44.6	90.07	13.31	98.36	13.66
VCI > PRI^b^	26	25.7	92.31	15.09	102.00	12.06
PRI > VCI^c^	30	29.7	93.57	11.54	100.20	13.69

*PSI* processing speed index, *FSIQ* full-scale intelligence quotient, *M* mean, *SD* standard deviation, *VCI* verbal comprehension index, *PRI* perceptual reasoning index

^a^ < 11 points difference

^b^ ≥ 11 points difference

^c^ ≥ 11 points difference

## Discussion

In this study, we found a significantly lower PSI relative to the norm and to the other indices of the WISC-IV in our sample of 101 school children with thoroughly diagnosed ASD. This PSI weakness was found independent of age (six to 16 years of age), general cognitive ability (WISC-IV FSIQ between 70 and 143), or IQ split (that is, a significant discrepancy between VCI and PRI in either direction). Notably, the PSI weakness was also independent of symptom severity. Although there was a small significant correlation between the ADOS communication domain and PSI (*r* = − 0.241) in the first place, the significance of this finding did not survive Bonferroni correction for multiple comparisons.

What is the clinical and practical relevance of this PSI weakness found in children with ASD? Previous studies pointed out that the PSI may be confounded with graphomotor speed due to the high demands its subtests (“Coding” and “Symbol search”) pose on the child with regard to fine motor skills and visual-motor coordination (Kenworthy et al., 2013; Oliveras-Rentas et al., 2012). This seems to be particularly true for children with ASD as (visuo-)motor difficulties have been found to occur with a higher probability in ASD compared to typically developing children (Kushki et al., 2011; Mayes & Calhoun, 2007). This point is substantiated by findings of lower scores on the Coding subtest of the PSI, which requires a higher degree of manual dexterity, than on the Symbol Search subtest, which is less demanding with regard to fine motor skills (Mayes & Calhoun, 2007, 2008; Nader et al., 2015). In addition, there are findings of normal speed of simple perceptual discrimination on a motor-free “inspection time task” in a sample of children with ASD (Wallace et al., 2009). These aspects should be kept in mind when interpreting PSI scores.

Nevertheless, as discussed above, the PSI confers valuable information about different aspects of a child’s educational and academic functioning, such as achievement scores in math, reading, and written expression, graphomotor ability, and the amount of hours spent with a special education assistant as well as executive functioning (Fry & Hale, 2000; Gordon et al., 2018; Grimm et al., 2015; Lundervold et al., 2011; Mayes & Calhoun, 2007; McAuley & White, 2011; Rohde & Thompson, 2007). The findings of the present study thus convey important practical implications for parents, teachers, and clinicians in the handling of ASD in educational and therapeutic settings. The results suggest that school children with “high-functioning” ASD (that is, without intellectual disability), independent of their age and intellectual ability, and—most noteworthily—independent of the severity of their ASD symptoms are in need of special support in educational contexts in order to compensate for the disadvantages conveyed by their PSI weakness. For example, supporting measures can take the form of additional breaks and time grants for exercises and examinations or printed handouts that spare children with ASD the necessity (and time) of copying and handwriting and enable them to track the teacher’s instructions. Considering the consistently reported positive association between children’s PSI scores and their academic functioning, such attempts at compensation for the disadvantages the processing speed (and potentially also graphomotor) weakness entails are highly warranted for the group of school children with ASD as a whole. In this regard, it is important to stress that our study did not link patient characteristics such as symptom severity and IQ split directly to achievement scores or grades. Future research should aim to further elucidate the association between these patient characteristics and academic achievement by linking these factors to more direct measures of achievement. Furthermore, when drawing inferences about girls with ASD, one should keep in mind that with 10 female patients in the sample, this group is—though represented according to the epidemiological gender distribution of ASD—underpowered in our study.

The lack of a control group consisting of typically developing children constitutes another limitation of our study. With regard to the association between PSI and age, the validity of our study is limited by its cross-sectional nature. Ultimately, it should be emphasized that the study focused on clinically noticeable, practically relevant influences on the PSI. There is a need for more research in order to unravel which cognitive mechanisms underlie the PSI weakness and how these children could be best supported in order to improve their overall life outcomes. Thus, future research should aim to investigate the respective contributions of manual dexterity, visuomotor integration, genuine speed of information processing and possible other factors to low PSI scores as well as its association with more subtle indicators of individual, social and academic performance in children with ASD.

Finally, in the investigation of ASD symptomatology using ADOS and ADI-R, symptom domains should be considered separately in the statistical analysis, rather than being merged into one score, as correlations between the respective domains of the two instruments may be insignificant or very small, as it was the case in this study.

## Conclusion

Low PSI scores in school children with “high-functioning” (FSIQ ≥ 70) ASD were found to be unrelated to age, general cognitive ability, VCI-PRI discrepancy, and most notably also independent of autistic symptoms. Considering the consistently reported positive association between PSI scores and various aspects of a child’s functioning in educational settings, the results of the present study suggest that school children with ASD in general, independent of their age, symptom severity, IQ split profile or general cognitive ability should be entitled to special educational assistance and measures that compensate for the disadvantages associated with their PSI weakness.
